# Direct comparison of SARS-CoV-2 variant specific neutralizing antibodies in human and hamster sera

**DOI:** 10.1038/s41541-024-00888-y

**Published:** 2024-05-18

**Authors:** Annika Rössler, Antonia Netzl, Ludwig Knabl, Samuel H. Wilks, Barbara Mühlemann, Sina Türeli, Anna Mykytyn, Dorothee von Laer, Bart L. Haagmans, Derek J. Smith, Janine Kimpel

**Affiliations:** 1 Institute of Virology, Department of Hygiene, Microbiology and Public Health, Medical University of Innsbruck, Peter-Mayr-Str. 4b, 6020 Innsbruck, Austria; 2 Center for Virology and Vaccine Research, Beth Israel Deaconess Medical Center, Harvard Medical School, Boston, MA USA; 3 University of Cambridge, Centre for Pathogen Evolution, Department of Zoology, Cambridge, UK; 4 Tyrolpath Obrist Brunhuber GmbH, Hauptplatz 4, 6511 Zams, Austria; 5 Institute of Virology, Charité – Universitätsmedizin Berlin, corporate member of Freie Universität Berlin, Humboldt-Universität zu Berlin, and Berlin Ins+titute of Health, 10117 Berlin, Germany; 6 German Centre for Infection Research (DZIF), partner site Charité, 10117 Berlin, Germany; 7 Viroscience Department, Erasmus Medical Center, Rotterdam, Netherlands

**Keywords:** Viral infection, SARS-CoV-2

## Abstract

Antigenic characterization of newly emerging SARS-CoV-2 variants is important to assess their immune escape and judge the need for future vaccine updates. To bridge data obtained from animal sera with human sera, we analyzed neutralizing antibody titers in human and hamster single infection sera in a highly controlled setting using the same authentic virus neutralization assay performed in one laboratory. Using a Bayesian framework, we found that titer fold changes in hamster sera corresponded well to human sera and that hamster sera generally exhibited higher reactivity.

## Introduction

Antigenic characterization is critical for tracking the evolution of severe acute respiratory syndrome coronavirus type 2 (SARS-CoV-2), assessing the immune escape of emerging SARS-CoV-2 variants, and judging the need for vaccine updates^1^. This requires the measurement of neutralizing antibody titers in cohorts representing the current population immunity to validate the need for a vaccine update. Additionally, single variant exposure sera are essential to assess the antigenic properties of the variant in the context of previously circulating variants, without confounding effects by immunity from previous infections. Multi-exposure sera are unsuitable for that purpose as multiple exposures generally increase cross-neutralization and thereby obscure the underlying antigenic relationships among variants^2,3^. By now, human single variant exposure sera against newly emerging SARS-CoV-2 variants are increasingly difficult to collect. However, antigenic characterization of virus variants can alternatively be performed using animal sera, as in the case of influenza virus^4^. Due to a lack of human single infection sera after decades of influenza virus circulation, ferrets are the standard organism for characterizing antigenic relationships of emerging influenza virus strains^4^. In SARS-CoV-2 research, sera from infected mice and hamsters have been widely used^5,6^. To overcome the scarcity of human single infection sera, bridging of human and animal data is an important task for future research and public health management of SARS-CoV-2.

Here, we directly compared neutralizing antibody titers from single exposure human and hamster sera. We selected four serum groups for this study: non-vaccinated individuals infected with the ancestral virus (614D/G, *n*_human_ = 10, *n*_hamster_ = 4), Delta (*n*_human_=7, *n*_hamster_ = 4), BA.1 Omicron (*n*_human_=17, *n*_hamster_ = 4), or BA.5 Omicron variant (n_human_=3, *n*_hamster_ = 3) (Supplementary Table 1). To minimize potential bias by different assays and interlaboratory variation, we analyzed neutralizing antibody titers for all samples in one laboratory using the same focus reduction neutralization assay (for staining of infected cells for human and hamster samples two different staining protocols were used) for 6 pre-Omicron (D614G, Alpha, Alpha-E484K, Beta, Gamma, Delta) and 4 Omicron (BA.1, BA.2, BA.5.3.2, XBB.1.5.1) variants (Supplementary Table 2). The variants and sera used had previously been published in independent studies in two different labs using different assays^2,7,8^. When comparing the published hamster data and the data we newly generated within this study we found that both data sets were largely in agreement except that we here measured lower BA.5 titers, which could be due to specific virus isolates of high or low reactivity. Additionally, the titers obtained in this study were roughly 4-fold lower than in the original publication.

**Fig. 1:**
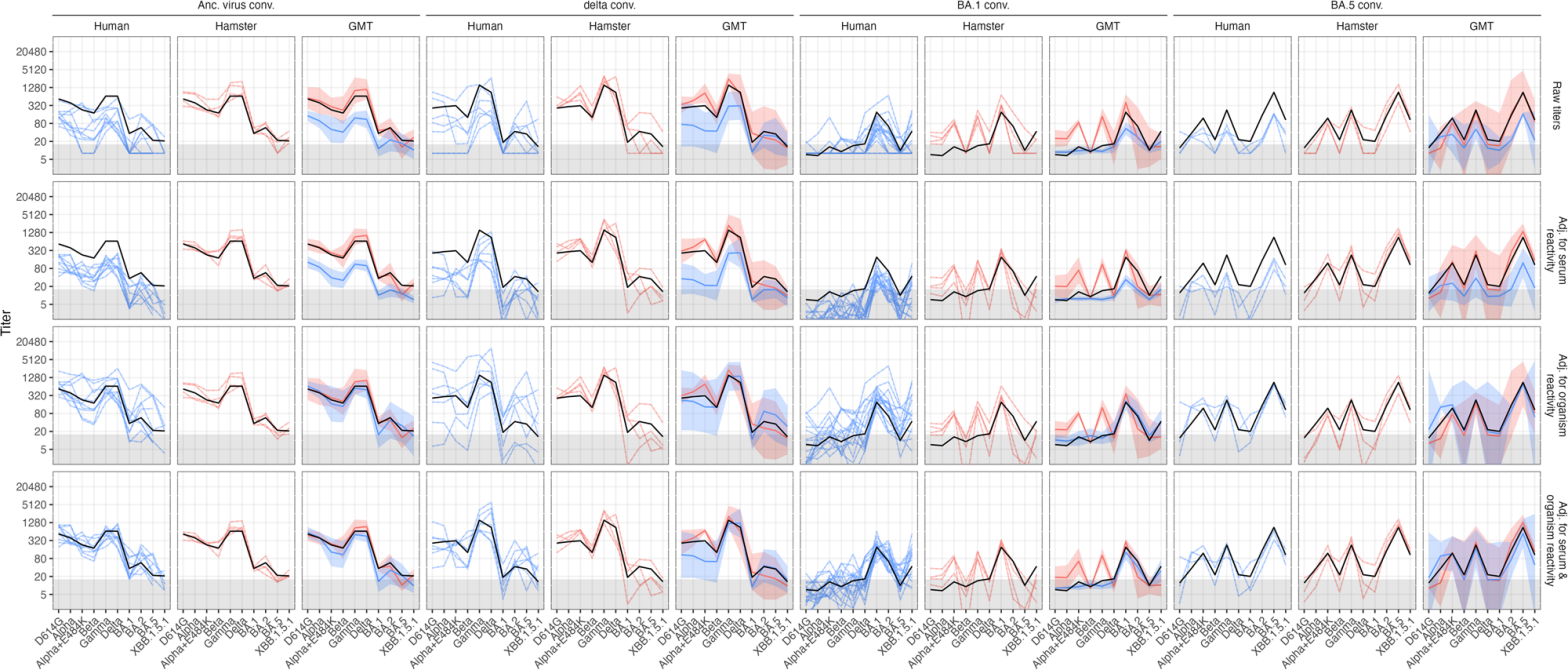
Comparison of neutralization titers in hamster and human single variant exposure sera. Human and hamster single variant exposure sera (human *n* = 10 ancestral, *n* = 7 delta, *n* = 17 BA.1, *n* = 3 BA.5; hamster *n* = 4 ancestral, *n* = 4 delta, *n* = 4 BA.1, *n* = 3 BA.5) were analyzed for neutralizing antibodies against D614G, alpha, alpha+E484K, beta, gamma, delta, BA.1, BA.2, BA.5, XBB.1.5.1 variants using a focus reduction assay and authentic virus variants. To control for titer variation due to different reactivities of individual sera and estimate species-specific effects, titers were estimated using a Bayesian framework (Supplementary Methods)^5^. The columns show human titers (blue), hamster titers (pink), and the GMT (geometric mean titers) ± +95% CI (confidence interval) as bold colored line and shaded area for each convalescent (conv.) serum group. The black line represents the estimated Geometric Mean Titer per serum group across organisms after adjusting for serum and organism effects. The rows show from top to bottom: Raw titers with titers <LOD (limit of detection ≤ 16 indicated by grey area) set to 8 (LOD/2), titers adjusted for individual serum reactivity variation, titers adjusted for organism reactivity differences, and titers adjusted for both individual serum and organism reactivities.

We found that while overall fold change trends for human and hamster titers were similar, variation between subjects in the same serum group was lower for hamster samples, and hamster titers were generally higher (Fig. 1, row 1). This was true for all serum groups. The higher variation in human samples might be explained by the natural variation between samples in this group in terms of genetic background, age, time since exposure, infecting dose, infecting virus etc. To control for this within and between serum magnitude difference, we employed a Bayesian framework, which was recently used to compare SARS-CoV-2 neutralization data across different laboratories and species^5^. In this framework, variations from the overall geometric mean titer for a variant in a serum group are attributed to different individual serum reactivities, reactivity differences due to species, and noise (Supplementary Methods). Using this statistical framework on our experimental data allowed us to quantify serum and organism-specific effects and obtain estimates for titers that fall below an assay’s limit of detection (LOD) (Fig. 1, rows 2-4).

Adjusting for serum reactivity effects reduced variation among individual samples as expected and showed that although titer magnitude may differ between individuals, the fold-changes (and consequently the relative titer differences between variants) were very similar and independent of titer magnitude (Fig. 1, row 2). We further found a systematic difference in titer magnitude between human and hamsters, and calculated hamster titers to be an average estimate of 4.9-fold higher than human titers (95% CI = 4.7-5.1; SD = 8.3, Fig. 1, row 2, magnitude adjusted in row 3). The human-hamster titer magnitude difference estimate of 3.1-fold higher hamster titers by Mühlemann et al.^5^ fell within the distribution’s interquartile range (Supplementary Figure 1D). After controlling for both within- and between-species titer magnitude effects, human and hamster titer fold-change trends were highly similar in the Ancestral virus convalescent (conv.) and BA.5 conv. serum groups, apart from lower titers against the Alpha variant in BA.5 conv. hamsters (Fig. 1, row 4). We further found that hamster BA.1 sera had higher titers against Alpha+E484K and Gamma than human sera. In Delta sera, too, hamsters exhibited higher titers against Alpha+E484K and Alpha than humans, but fold-change trends were otherwise similar. To summarize, fold-change patterns are remarkably consistent across humans and hamsters, except for the few differences described above.

Mühlemann et al. introduced a systematic framework to compare SARS-CoV-2 antigenic data from different laboratories, generated without sharing of sera or variants and using different assays^5^. Here, we compared data obtained in a highly controlled setting: Human and hamster sera were measured in a single laboratory in the same assay and with the same virus stock. Mühlemann et al.’s framework recommends the evaluation of antigenic data on four levels: Titer magnitude, titer fold-changes, immunodominance patterns, and antigenic cartography^5^. Although the controlled nature of our study comes with the limitation that we did not have serum groups available from both species with known immunodominance changes, such as Beta convalescent sera^5,9^, and lacked single-exposure sera from diverse variants to construct well-triangulated antigenic maps (Supplementary Figure 2), it allowed for exact comparison of titer magnitude and fold-change trends. Our data indicate that titer fold changes in hamster sera correspond well to human sera, and that titers correspond well after adjusting the data for the higher reactivity generally seen in the hamster. The higher reactivity in hamster sera will consequently not influence the antigenic relationships determined by the assay, but rather provide benefit as a greater range of neutralization can be measured before values fall below an assay’s limit of detection. In the context of antigenic cartography, this greater detection range can be especially useful as it permits longer-range triangulation and consequently increases map resolution^4,10^. In summary, for the sera raised, variants tested, and assay used here, sera from infected hamsters are a good surrogate for human sera for the antigenic characterization of SARS-CoV-2 variants and overcome the limitation of collecting human single variant exposure sera for newly emerging virus variants. It will be important, however, to continue to test the suitability of hamster sera as a model for single-exposure human sera against newer variants.

## Methods

### Human samples

Sera were collected from 37 individuals after SARS-CoV-2 single variant exposure. In more detail, we analyzed the samples from individuals after infection with ancestral (*n* = 10), Delta (*n* = 7) or Omicron BA.1 (*n* = 17) or BA.5 (*n* = 3) variant and study cohorts are characterized in Supplementary Table 1. The ethics committee (EC) of the Medical University of Innsbruck has approved sample collection with EC numbers: 1100/2020, 1111/2020, 1330/2020, 1064/2021, 1093/2021, 1168/2021, 1191/2021, 1197/2021, and 1059/2022. Written informed consent has been obtained from study participants.

### Animal samples

Female Syrian golden hamster sera was obtained as described previously^7,8^. Briefly, animals were inoculated intranasally with 1 × 10^5^ plaque-forming units of ancestral (*n* = 4) or Omicron BA.5 (*n* = 3), or 5 × 10^4^ of Delta (*n* = 4) or Omicron BA.1 (*n* = 4). Omicron BA.5 animals were euthanized at 21 days postinfection, whereas all other animals were euthanized at 26 days postinfection, at which point serum was collected. Animals were monitored daily, and at the first sign of disease, observation frequency was increased. Animals were euthanized if one of the following points was reached; animal no longer eats or drinks, loss of more than 15% body weight over two days or more than 25% of body weight loss compared to the start of the experiment, more than moderate respiratory distress whereby respiratory problems (convulsive breathing, contraction of the flanks) or breathlessness are considered a human end-point criterion, behavioral and movement patterns deviate significantly from routine. No animals reached the above criteria, therefore all animals were euthanized based on the predetermined time-point (of 21 or 26 days) considered to allow seroconversion to reach homologous neutralizing titers of approximately 1:1000 or higher. Animals were euthanized by cardiac puncture under isoflurane anesthesia and cervical dislocation. This research was in compliance with the Dutch legislation for the protection of animals used for scientific purposes (2014, implementing EU Directive 2010/63). This research was conducted either at Erasmus MC (approved OLAW Assurance no. A5051-01, study protocol no. 17-4312 approved by institutional Animal Welfare Body) or at Viroclinics Biosciences B.V., Viroclinics Xplore (license number AVD27700202114492-WP35).

### Neutralization assay

Human and hamster samples were tested for neutralization against a panel of 10 authentic SARS-CoV-2 isolates, which included several pre-Omicron variants (D614G, Alpha, Alpha with additional E484K mutation, Beta, Gamma and Delta), three Omicron variants (BA.1, BA.2 and BA.5) as well as a recombinant lineage (XBB.1.5.1). Details on used virus isolates are shown in Supplementary Table 2. To analyze neutralization titer, we performed a focus-forming assay as previously described^2^. Therefore, four-fold dilutions of heat-inactivated sera were incubated with SARS-CoV-2 isolates for 1 h at 37°C and subsequently transferred to Vero-cells overexpressing TMPRSS2 and ACE2. The virus/sera mix was replaced by fresh medium 2 h after infection and cells were fixed with absolute ethanol further 8 h later. Infected cells were visualized by immunofluorescence staining. Results of the human samples were developed using a SARS-CoV-2 convalescent plasma as primary and a goat anti-human IgG Alexa Fluor 488 secondary antibody (1:1000 diluted, ThermoFisher Scientific #A48276)^2^. For hamster samples a SARS-CoV-2 Nucleocapsid antibody (1:500 diluted, SinoBiological #40143-T62) followed by an goat anti-rabbit IgG Alexa Fluor 488 antibody (1:2000 diluted, ThermoFisher Scientific #A32731) was used^8^. Infected cells were counted using an immunospot reader and continuous neutralization titers (IC_50_) were calculated by non-linear regression (GraphPad Prism Software 9.0.1, Inc., La Jolla, CA, USA). Titers ≥16,384 were set to 16,384. Neutralization titers ≥16 were considered positive and negative titers were set to half the detection limit, i.e. 8. For Bayesian modelling and antigenic cartography (Supplementary Figure 2), titers <16 were set to “<16”.

### Titer adjustments

A recent study comparing SARS-CoV-2 neutralization data from different assays, species and laboratories found that reactivity patterns were similar, but titer magnitude varied by species^5^. Neutralization titers measured in distinct laboratories can differ due to various reasons, the most obvious being the assay performed such as authentic vs. pseudotyped virus and the cell type used in the assay. Hence, comparing these values from different sources without adjusting for said differences could lead to the faulty conclusion that titers differ systematically. To not only control for this but also quantify these effects, Mühlemann et al.^5^ developed a Bayesian modelling framework in which titer magnitude differences due to individual serum reactivities, differences across organisms and across laboratories are explicitly accounted for.

In this study, all titers were measured in the same laboratory and with the same assay (for staining of infected cells for human and hamster samples two different staining protocols were used) as the question of interest was whether systematic differences between hamster and human neutralizing antibody responses to SARS-CoV-2 infection exist. To target this question, the framework by Mühlemann et al.^5^ was adjusted by omitting the parameter for laboratory-specific effects. Individual serum reactivity effects were controlled for as these could occur due to varying times since infection or severity of infection in the humans, or high/low responders. Each titer is modelled as a combination of geometric mean titer per variant and serum group, a reactivity effect of each individual serum, and a species-specific magnitude difference.

1 $${{logtiter}}_{{ijm}}={{serumGroupGMT}}_{{iJ}}+{{serumEffect}}_{j}+{{organismEffect}}_{m}+{\varepsilon }_{{ij}}$$

In Eq. 1, the *serumGroupGMT* refers to the log2 titer of antigen *i* in serum group *J*, the *serumEffect* corresponds to the reactivity bias of serum *j*, the *organismEffect* to the reactivity bias of organism *m*, and log2 normally distributed noise $\varepsilon$ for each measurement, where the standard deviation is assumed to differ between organisms.

The model was based on the cmdstanr model used by Mühlemann et al.^5^ (R version 4.2.2^11^, cmdstanr version 0.5.3^12^), and priors for the standard deviation parameters were chosen based on their values (inverse gamma distribution, shape = 3, scale = 1.5). For modelling both serum and organism reactivity effects, the following distributions were used: serumGroupGMT: N(3, 20), serumEffect: N(0, 10), organismEffect: N(0, 2). For modelling only serum or organism effects (Supplementary figures 1-3), the standard deviation of the excluded effect was set to 1e-3 to penalize any deviation from the mean 0. The mean of the posterior distributions when modelling both organism and serum reactivity was used to adjust the raw titers (Supplementary Figure 1). cmdstanr’s sampling, rather than its optimization function, was used as a comparison of values revealed a small difference between posterior means and optimized values (Supplementary Figures 1, 3-4). This may happen when the posterior distribution is not convex and the optimization algorithm gets stuck in a local optimum instead of a global one. The models were run for 3000 iterations with 1000 warmup iterations on 4 parallel chains and a maximum treedepth of 20.

Shinystan’s launch_shinystan function was used for model diagnostics^13^. R-hat is a measure of MCMC chain convergence which compares between- and within-chain model parameters. Values larger than 1 indicate that the independently sampling chains did not mix well and did not converge to a common distribution. Models with values above 1.05 should be discarded^14^. All parameter R-hat values were below 1.05 in the model for only serum reactivity and the model for both serum and organism reactivity. In the model for organism reactivity only, some parameters had R-hat values above the cut-off, indicating that the model should be further optimized for modelling only organism reactivity magnitude differences. As the primary aim of our study was to compare organism and serum effects simultaneously and this was only supplementary analysis to demonstrate that the simultaneous approach was more suitable, we decided to report the results as obtained by the non-optimized organism reactivity only-model and their caveats.

The effective sample size (ESS) gives the number of independent, not auto-correlated draws and holds information on parameter uncertainty. GMTs per antigen and serum group, serum and organism reactivity effects had an effective sample size >10% of actual sample size in the serum reactivity only and combined model but ESS was below that for GMTs and organism reactivity effects in the organism reactivity only model, indicating that this model should be further optimized if used to make inferences.

All code can be found in the manuscript’s GitHub repository^15^: https://github.com/acorg/roessler_netzl_et_al2023a.git

### Reporting summary

Further information on research design is available in the Nature Research Reporting Summary linked to this article.

### Supplementary information

Supplementary Materials

Reporting Summary

## Data Availability

All data is publicly available in the manuscript’s GitHub repository^15^ (https://github.com/acorg/roessler_netzl_et_al2023a.git).

## Code availability

All code is publicly available in the manuscript’s GitHub repository^15^ (https://github.com/acorg/roessler_netzl_et_al2023a.git).
