# Supplementary material for: Direct comparison of SARS-CoV-2 variant specific neutralizing antibodies in human and hamster sera
Source: NPJ Vaccines. 2024 May 18;9:85. doi: 10.1038/s41541-024-00888-y (PMC11102554; doi:10.1038/s41541-024-00888-y)
Supplement: Supplementary file 1 — Supplementary Materials [file 41541_2024_888_MOESM1_ESM.pdf]

Supplementary Figures

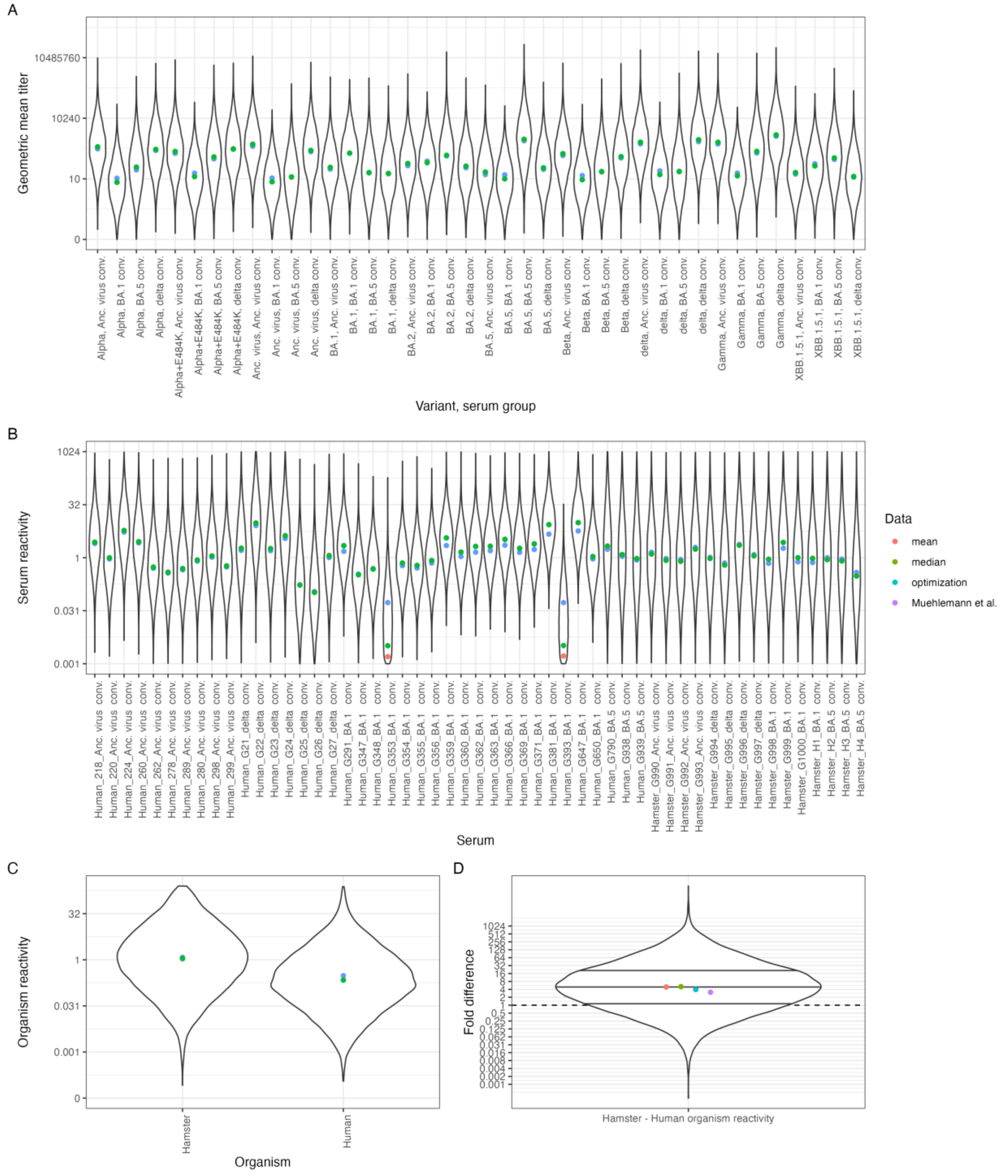

Supplementary Figure 1. Posterior distribution when modelling serum and organism reactivity. Posterior distributions of draws for **A)** variant geometric mean titers, **B)** serum reactivities, and **C)** organism reactivities are shown. **D)** Shows the difference between hamster and human organism reactivity with solid horizontal bars marking the 1st (1.2), 2nd (5) and 3rd (20.9) quartile from top to bottom and the dashed reference line indicating no fold difference. The mean of the distribution is shown in pink, the median in green and the optimized value in blue. The value reported by Mühlemann *et al.* [1] is given in **D)** in purple. The reactivity corresponds to a fold change of titers against all antigen variants.

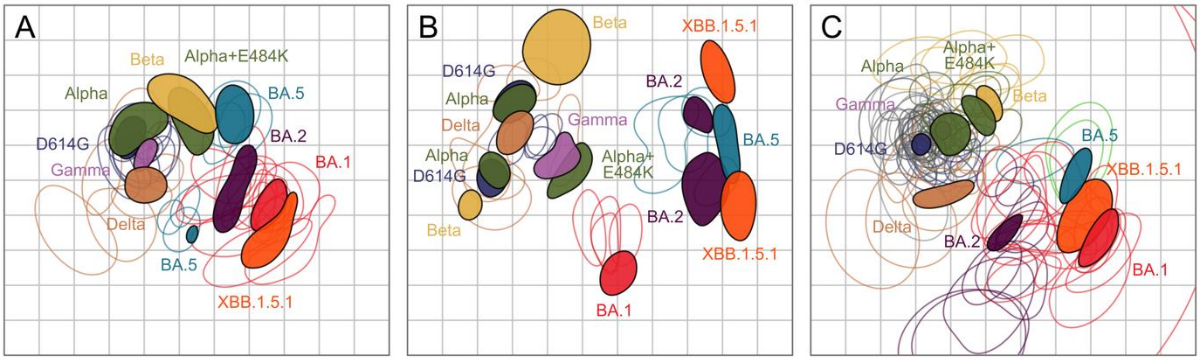

Supplementary Figure 2. Positional uncertainty in human and hamster antigenic maps. A) Human and **B)** hamster antigenic maps were constructed in Racmacs [2] (v 1.1.35) with 1000 optimizations in 2 dimensions. **C)** Human map with additional serum groups (two dose Ancestral variant vaccinated, Beta convalescent, Alpha convalescent, BA.2 convalescent) subset to variants in this comparison (originally published by Rössler *et al.* [3]). Positional variation of human and hamster sera was determined by Bayesian bootstrapping, where sera and antigen measurements are weighted by random draws from a Dirichlet distribution, such that individual sera and antigens contribute to the map optimization with different weights. 500 bootstrap repeats were performed with 1000 optimizations each. The colored regions mark 68 % (one standard deviation) of the positional variation for each variant (filled shapes) and sera (open shapes). Multiple shapes per variant, as seen in the human map for BA.5 and in the hamster map for D614G, Alpha, BA.2 and XBB.1.5.1, indicate that multiple optima exists and that the map is not well triangulated. Additional serum groups increase map triangulation and positional certainty as shown in **C.**

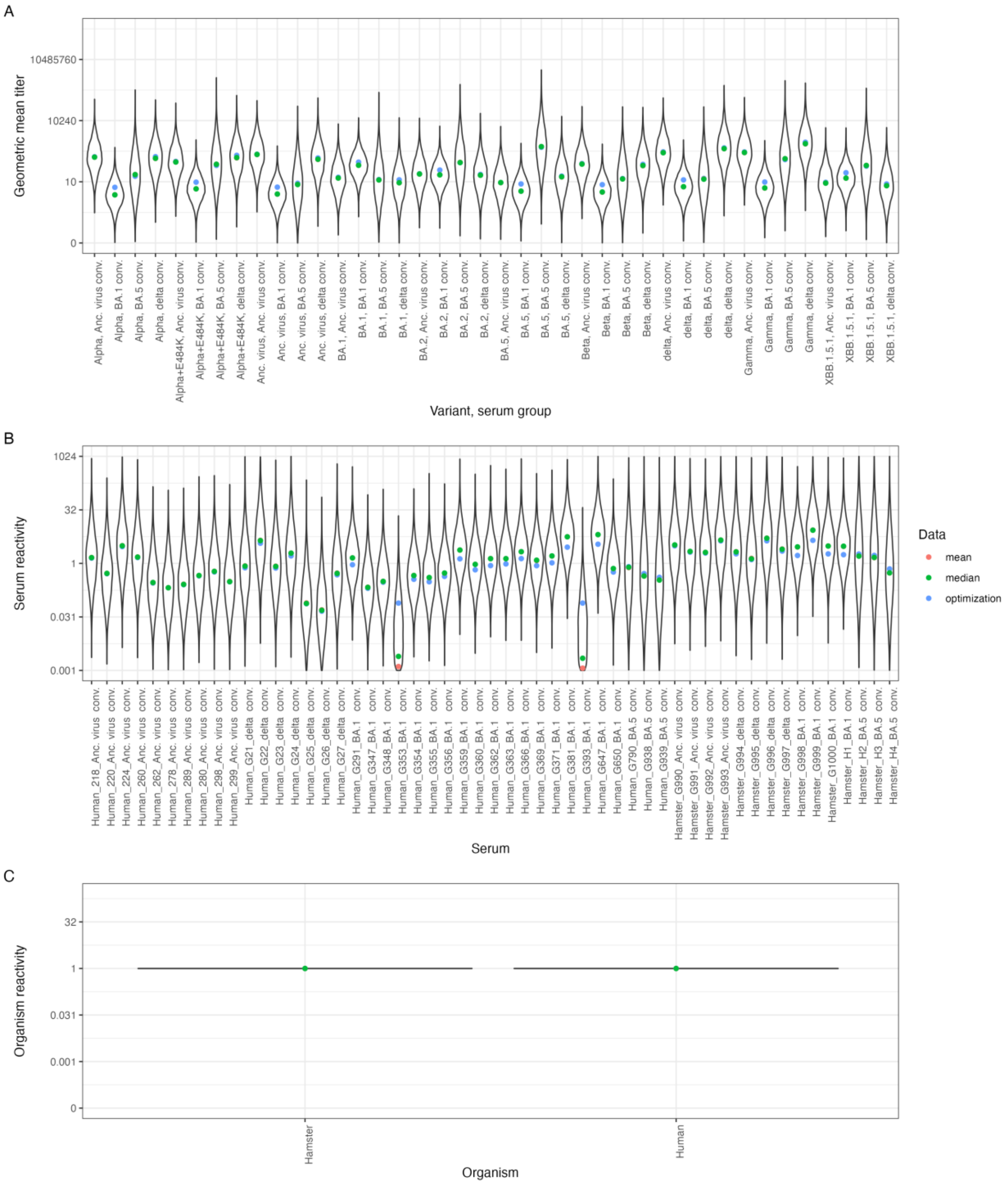

Supplementary Figure 3. Posterior distribution when modelling only serum reactivity. Posterior distributions of draws for **A)** variant geometric mean titers, **B)** serum reactivities, and **C)** organism reactivities are shown. The mean of the distribution is shown in pink, the median in green and the optimized value in blue. The reactivity corresponds to a fold change of titers against all antigen variants.

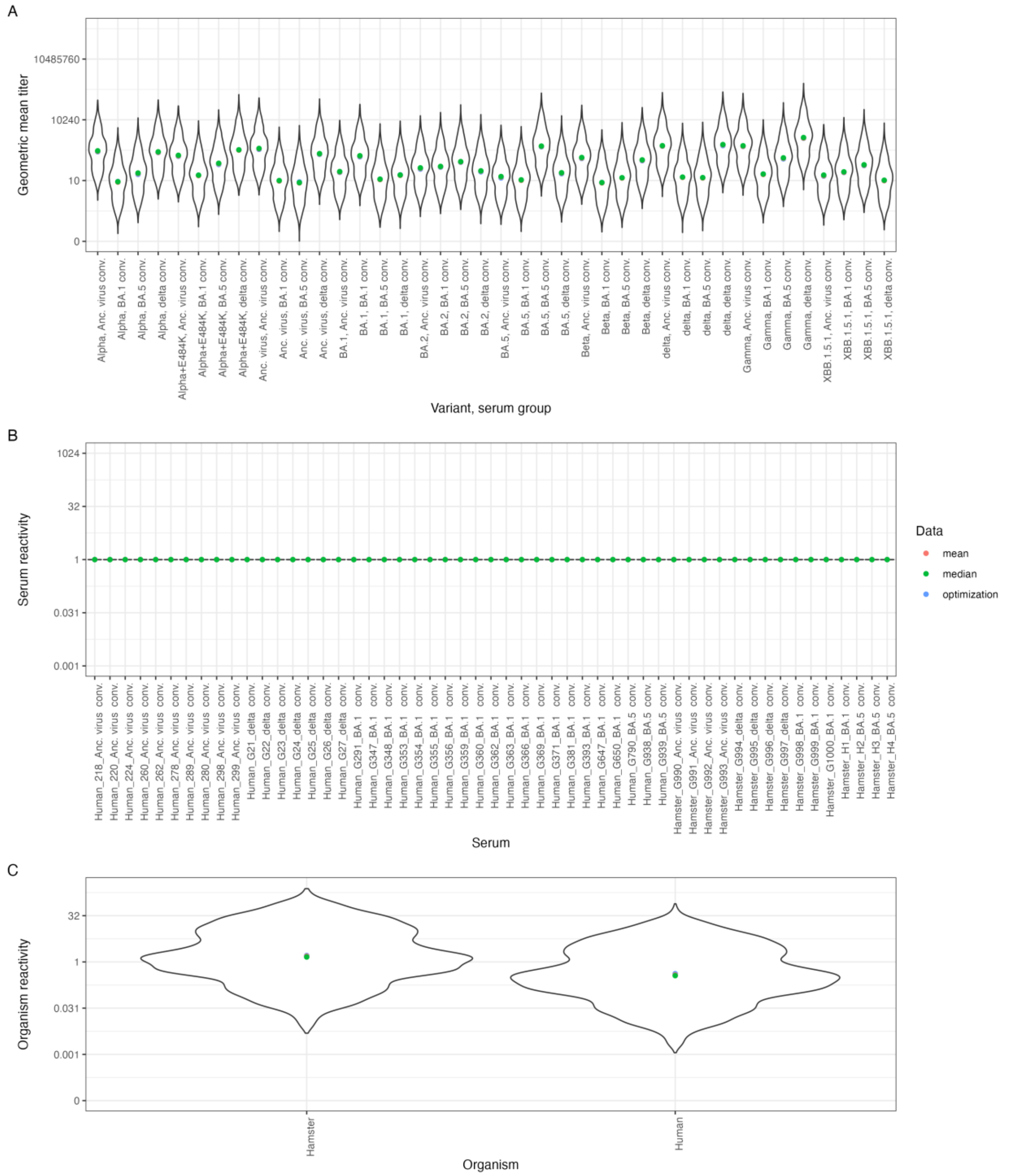

Supplementary Figure 4. Posterior distribution when modelling only organism reactivity. Posterior distributions of draws for **A)** variant geometric mean titers, **B)** serum reactivities, and **C)** organism reactivities are shown. The mean of the distribution is shown in pink, the median in green and the optimized value in blue. The reactivity corresponds to a fold change of titers against all antigen variants.

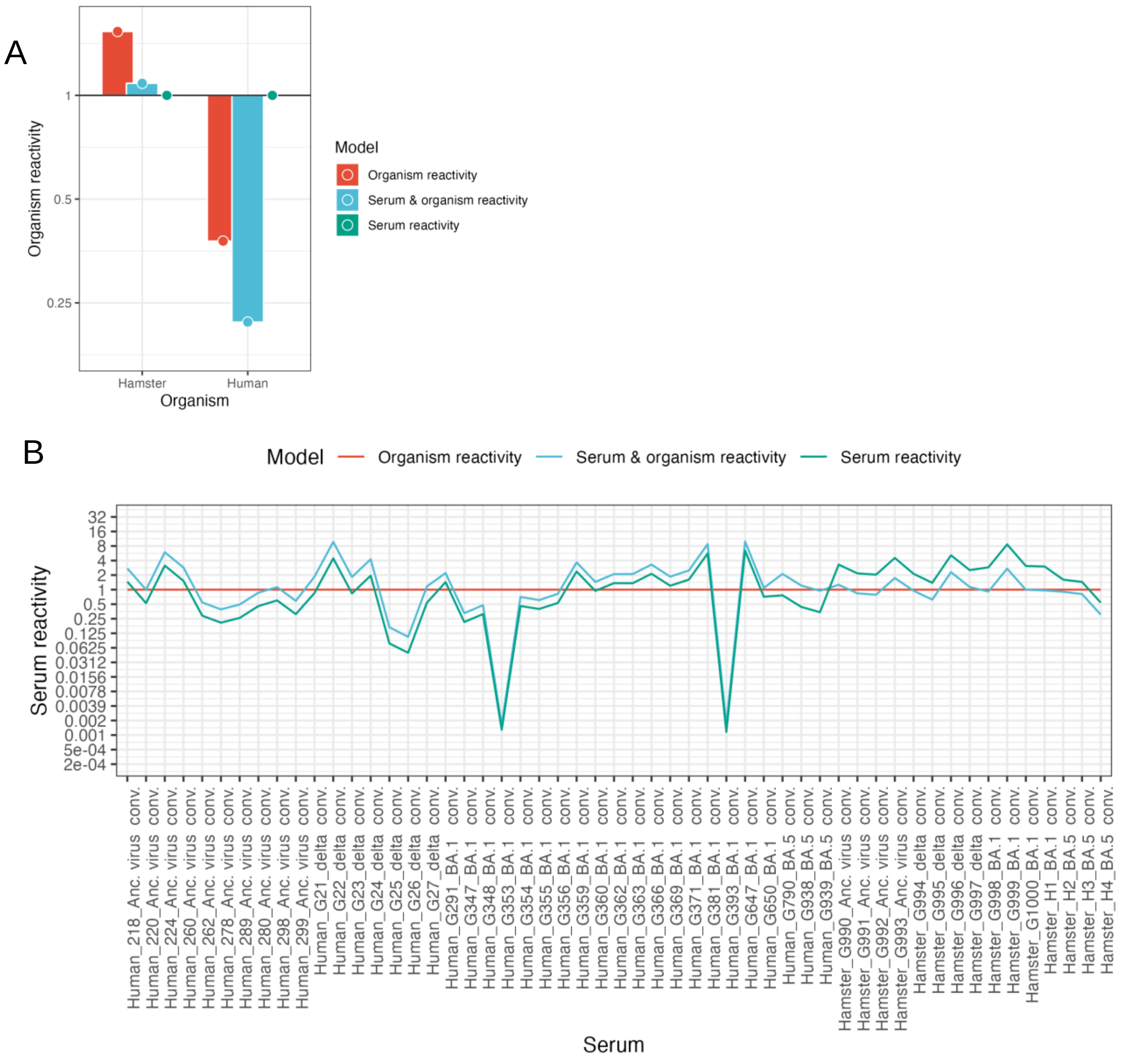

Supplementary Figure 5. Mean organism and serum reactivity effects. The mean of the sampled posterior distributions for **A)** organism reactivities and **B)** serum reactivities are shown. The red color shows mean values when only organism reactivities were estimated, the green when only serum reactivities were estimated, and the light blue when both organism. The reactivity corresponds to a fold change of titers against all antigen variants.

Supplementary Table 1. Sample characteristics

Species	Exposed Virus Variant	Number	Time since positive PCR/infection[#]
Human	Ancestral	10*	2 – 6 weeks [§]
Human	Delta	7	5 weeks (4 - 18)
Human	BA.1 Omicron	17	2 weeks (1.7 - 2.4)
Human	BA.5 Omicron	3	3 weeks (2.7 - 3)
Hamster	Ancestral	4	26 days
Hamster	Delta	4	26 days
Hamster	BA.1 Omicron	4	26 days
Hamster	BA.5 Omicron	3	21 days

*5 of the 10 human ancestral variant exposed sera and 2 of the 17 human BA.1 exposed sera were not tested against XBB.1.5.1 due to limited sample volume; [#]Intervals between positive PCR and blood collection for the different human serum groups were expressed by median and interquartile range (IQR) in weeks when exact intervals were known. Interval between infection and sample collection for the hamster sera are given in days; [§]For the human ancestral convalescent samples, intervals were given as period in weeks, as exact intervals were not known.

Supplementary Table 2. Virus variants used.

Isolate ID*	Variant	Pango lineage[§]	WHO nomenclature	GISAID ID
B86.2	D614G (ancestral)	B.1.177	D614G	EPI_ISL_3305837
C63.1	Alpha	B.1.1.7	Alpha	EPI_ISL_3277382
C79.2	Alpha-E484K	B.1.1.7 (E484K)	Alpha (E484K)	EPI_ISL_3277383
C24.1	Beta	B.1.351	Beta	EPI_ISL_17528983
D94	Gamma	P.1.1	Gamma	EPI_ISL_2095177
D27	Delta	B.1.617.2	Delta	EPI_ISL_2290769
E16.1	BA.1 Omicron	BA.1	Omicron BA.1	EPI_ISL_17528984
E65.1	BA.2 Omicron	BA.2	Omicron BA.2	EPI_ISL_12486408
E73.1	BA.5 Omicron	BA.5.3.2	Omicron BA.5	EPI_ISL_13666092
G37.2	XBB.1.5 Omicron	XBB.1.5.1	XBB.1.5.1	EPI_ISL_17077093

*Internal name of isolate; [§]determined using UShER (https://genome.ucsc.edu/cgi-bin/hgPhyloPlace) on 24.03.2023

References

1 Mühlemann, B. *et al.* Comparative Analysis of SARS-CoV-2 Antigenicity across Assays and in Human and Animal Model Sera. *bioRxiv*, 2023.2009.2027.559689, doi:10.1101/2023.09.27.559689 (2023).

2 Wilks, S. *Racmacs: R Antigenic Cartography Macros*, <https://acorg.github.io/Racmacs/index.html> (

3 Rössler, A. *et al.* Characterizing SARS-CoV-2 neutralization profiles after bivalent boosting using antigenic cartography. *Nat Commun* **14**, 5224, doi:10.1038/s41467-023-41049-4 (2023).
