# Supplementary material for: Direct comparison of SARS-CoV-2 variant specific neutralizing antibodies in human and hamster sera
Source: NPJ Vaccines. 2024 May 18;9:85. doi: 10.1038/s41541-024-00888-y (PMC11102554; doi:10.1038/s41541-024-00888-y)
Supplement: Supplementary file 2 — Reporting Summary [file 41541_2024_888_MOESM2_ESM.pdf]

nature portfolio

Corresponding author(s): Derek J. Smith & Janine Kimpel

Last updated by author(s): Mar 8, 2024

Reporting Summary

Nature Portfolio wishes to improve the reproducibility of the work that we publish. This form provides structure for consistency and transparency in reporting. For further information on Nature Portfolio policies, see our Editorial Policies and the Editorial Policy Checklist.

Statistics

For all statistical analyses, confirm that the following items are present in the figure legend, table legend, main text, or Methods section.

n/a	Confirmed	
☐	☒	The exact sample size (*n*) for each experimental group/condition, given as a discrete number and unit of measurement
☐	☒	A statement on whether measurements were taken from distinct samples or whether the same sample was measured repeatedly
☒	☐	The statistical test(s) used AND whether they are one- or two-sided<br>*Only common tests should be described solely by name; describe more complex techniques in the Methods section.*
☒	☐	A description of all covariates tested
☒	☐	A description of any assumptions or corrections, such as tests of normality and adjustment for multiple comparisons
☐	☒	A full description of the statistical parameters including central tendency (e.g. means) or other basic estimates (e.g. regression coefficient) AND variation (e.g. standard deviation) or associated estimates of uncertainty (e.g. confidence intervals)
☒	☐	For null hypothesis testing, the test statistic (e.g. *F*, *t*, *r*) with confidence intervals, effect sizes, degrees of freedom and *P* value noted<br>*Give P values as exact values whenever suitable.*
☐	☒	For Bayesian analysis, information on the choice of priors and Markov chain Monte Carlo settings
☒	☐	For hierarchical and complex designs, identification of the appropriate level for tests and full reporting of outcomes
☒	☐	Estimates of effect sizes (e.g. Cohen's *d*, Pearson's *r*), indicating how they were calculated

Our web collection on statistics for biologists contains articles on many of the points above.

Software and code

Policy information about availability of computer code

Data collection	Infected cells were counted using an an ImmunoSpot S6 Ultra-V reader and CTL analyzer BioSpot® 5.0 software (CTL Europe GmbH, Bonn, Germany) and continuous neutralization titers (IC50) were calculated by non-linear regression (GraphPad Prism Software 9.0.1, Inc., La Jolla, CA, USA).
Data analysis	All analyses were performed in R version 4.2.2 (2022-10-31). All code can be found in the manuscript's GitHub repository : https://github.com/acorg/roessler_netzl_et_al2023a.git

For manuscripts utilizing custom algorithms or software that are central to the research but not yet described in published literature, software must be made available to editors and reviewers. We strongly encourage code deposition in a community repository (e.g. GitHub). See the Nature Portfolio guidelines for submitting code & software for further information.

Data

Policy information about availability of data

All manuscripts must include a data availability statement. This statement should provide the following information, where applicable:

- Accession codes, unique identifiers, or web links for publicly available datasets
- A description of any restrictions on data availability
- For clinical datasets or third party data, please ensure that the statement adheres to our policy

Data availability

All data is publicly available in the manuscript's GitHub repository (https://github.com/acorg/roessler_netzl_et_al2023a.git).

Code availability
All code is publicly available in the manuscript's GitHub repository (https://github.com/acorg/roessler_netzl_et_al2023a.git).

Research involving human participants, their data, or biological material

Policy information about studies with human participants or human data. See also policy information about sex, gender (identity/presentation), and sexual orientation and race, ethnicity and racism.

Reporting on sex and gender	Data on human participants were reported in an earlier study, Rössler et al. 2023, Nature Communications, DOI: 10.1038/s41467-023-41049-4
Reporting on race, ethnicity, or other socially relevant groupings	Data on human participants were reported in an earlier study, Rössler et al. 2023, Nature Communications, DOI: 10.1038/s41467-023-41049-4
Population characteristics	Sera from non-vaccinated participants after a single infection with SARS-CoV-2 were used.
Recruitment	Data on human participants were reported in an earlier study, Rössler et al. 2023, Nature Communications, DOI: 10.1038/s41467-023-41049-4
Ethics oversight	The ethics committee (EC) of the Medical University of Innsbruck has approved sample collection with EC numbers: 1100/2020, 1111/2020, 1330/2020, 1064/2021, 1093/2021, 1168/2021, 1191/2021, 1197/2021, and 1059/2022.

Note that full information on the approval of the study protocol must also be provided in the manuscript.

Field-specific reporting

Please select the one below that is the best fit for your research. If you are not sure, read the appropriate sections before making your selection.

☒ Life sciences ☐ Behavioural & social sciences ☐ Ecological, evolutionary & environmental sciences

For a reference copy of the document with all sections, see nature.com/documents/nr-reporting-summary-flat.pdf

Life sciences study design

All studies must disclose on these points even when the disclosure is negative.

Sample size	No sample size calculation was performed. For the human sera we used data from a previous study (Rössler et al. 2023, Nature Communications, DOI: 10.1038/s41467-023-41049-4) and selected matching serum groups for infected hamsters. Number of samples analyzed per group is reported in Supplementary Table 1.
Data exclusions	No data were excluded.
Replication	We previously decribed the reproducibility of the here used neutralization assay (Riepler et al. 2020, Vaccines (Basel)). Each sample was analyzed once in the assay and experiments were not replicated.
Randomization	Not applicable as participants were allocated to the different groups due their infection history.
Blinding	No blinding of samples during analysis in neutralization assays was performed. However, the number of infected cells in the neutralization assay were automatically counted using an immunospot reader removing potential bias in data aquisition. Additionally, manual quality control to remove fibres etc. has been done blinded. IC50 titers were calculated automatically using Graphpad Prism.

Reporting for specific materials, systems and methods

We require information from authors about some types of materials, experimental systems and methods used in many studies. Here, indicate whether each material, system or method listed is relevant to your study. If you are not sure if a list item applies to your research, read the appropriate section before selecting a response.

Materials & experimental systems

n/a	Involved in the study
☐	☒ Antibodies
☐	☒ Eukaryotic cell lines
☒	☐ Palaeontology and archaeology
☐	☒ Animals and other organisms
☒	☐ Clinical data
☒	☐ Dual use research of concern
☒	☐ Plants

Methods

n/a	Involved in the study
☒	☐ ChIP-seq
☒	☐ Flow cytometry
☒	☐ MRI-based neuroimaging

Antibodies

Antibodies used	For human samples, a SARS-CoV-2 convalescent serum 1:1,000 diluted as primary antibody followed by goat anti-human Alexa Fluor Plus 488-conjugated secondary antibody, 1:1,000 diluted; Ref. A48276, Invitrogen, Thermo Fisher Scientific, Vienna, Austria was used. For hamster samples a SARS-CoV-2 Nucleocapsid antibody (1:500 diluted, SinoBiological #40143-T62) followed by an goat anti-rabbit IgG Alexa Fluor 488 antibody (1:2,000 diluted, ThermoFisher Scientific #A32731) was used .
Validation	No antibody validation has been performed during this study. Primary antibody has been used and validated for a previous study (Riepler et al. 2020, Vaccines (Basel) 9). The secondary antibody is commercially available and binds according to manufacturers specification to human IgG, but does not bind to non-immunoglobulin human serum proteins or serum proteins/IgG from bovine, mouse, and rabbit (https://www.thermofisher.com/order/genome-database/dataSheetPdf?producttype=antibody&productsubtype=antibody_secondary&productId=A48276&version=326).

Eukaryotic cell lines

Policy information about cell lines and Sex and Gender in Research

Cell line source(s)	In-house generated Vero (African green monkey kidny cell line) derivate stably overexpressing ACE2 and TMPRSS2
Authentication	no cell line authentication
Mycoplasma contamination	Cells were tested negative for mycoplasma
Commonly misidentified lines (See ICLAC register)	No commonly missidentified cell line has been used for this study.

Animals and other research organisms

Policy information about studies involving animals; ARRIVE guidelines recommended for reporting animal research, and Sex and Gender in Research

Laboratory animals	Syrian golden hamster
Wild animals	no wild animals used
Reporting on sex	For this study only female animals were used for antigenic characterization of SARS-CoV-2 variants. We analyzed fold-changes in neutralization activity within each serum for different virus variants. While absolute titers of neutralizing antibodies may vary between male and female animals, we do not assume that neutralization pattern in an invididual serum should differ depending on the sex of the animals.
Field-collected samples	The study did not involve samples collected from the field.
Ethics oversight	This research was in compliance with the Dutch legislation for the protection of animals used for scientific purposes (2014, implementing EU Directive 2010/63). This research was conducted either at Erasmus MC (approved OLAW Assurance no. A5051-01, study protocol no. 17-4312 approved by institutional Animal Welfare Body) or at Viroclinics Biosciences B.V., Viroclinics Xplore (license number AVD27700202114492-WP35).

Note that full information on the approval of the study protocol must also be provided in the manuscript.

Plants

Seed stocks	*Report on the source of all seed stocks or other plant material used. If applicable, state the seed stock centre and catalogue number. If plant specimens were collected from the field, describe the collection location, date and sampling procedures.*
Novel plant genotypes	*Describe the methods by which all novel plant genotypes were produced. This includes those generated by transgenic approaches, gene editing, chemical/radiation-based mutagenesis and hybridization. For transgenic lines, describe the transformation method, the number of independent lines analyzed and the generation upon which experiments were performed. For gene-edited lines, describe the editor used, the endogenous sequence targeted for editing, the targeting guide RNA sequence (if applicable) and how the editor was applied.*
Authentication	*Describe any authentication procedures for each seed stock used or novel genotype generated. Describe any experiments used to assess the effect of a mutation and, where applicable, how potential secondary effects (e.g. second site T-DNA insertions, mosiacism, off-target gene editing) were examined.*
